# A pharmacokinetics study of proposed bevacizumab biosimilar MYL-1402O vs EU-bevacizumab and US-bevacizumab

**DOI:** 10.1007/s00432-021-03628-0

**Published:** 2021-04-17

**Authors:** Matthew Hummel, Tjerk Bosje, Andrew Shaw, Mark Shiyao Liu, Abhijit Barve, Mudgal Kothekar, Mark A. Socinski, Cornelius F. Waller

**Affiliations:** 1 Viatris Inc, Morgantown, WV USA; 2 PRA Health Sciences, Groningen, The Netherlands; 3 Viatris Inc, Canonsburg, PA USA; 4 Biocon Research Ltd (Now With Sun Pharma Advanced Research Company, Mumbai, India), Bangalore, India; 5 AdventHealth Cancer Institute, Orlando, FL USA; 6 Department of Haematology, Oncology and Stem Cell Transplantation, University Medical Centre Freiburg and Faculty of Medicine, University of Freiburg, Hugstetter Str. 55, 79106 Freiburg, Germany

**Keywords:** Bioequivalence, Cancer, Monoclonal antibody, Pharmacokinetics, Phase 1

## Abstract

**Purpose:**

Bevacizumab is a recombinant humanized monoclonal antibody that inhibits vascular endothelial growth factor-specific angiogenesis in some cancers. MYL-1402O is a proposed bevacizumab biosimilar.

**Methods:**

The primary objective of this single-center, randomized, double-blind, three-arm, parallel-group, phase 1 study in healthy male volunteers was to evaluate bioequivalence of MYL-1402O to EU and US-reference bevacizumab, and EU-reference bevacizumab to US-reference bevacizumab. The primary pharmacokinetic parameter was area under the serum concentration–time curve from 0 extrapolated to infinity (AUC_0–∞_). Pharmacokinetic parameters were analyzed using general linear models of analysis of variance. Secondary endpoints included safety and tolerability.

**Results:**

Of 111 enrolled subjects, 110 were included in the pharmacokinetic analysis (MYL-1402O, *n* = 37; EU-reference bevacizumab, *n* = 36; US-reference bevacizumab, *n* = 37). Bioequivalence was demonstrated between MYL-1402O and EU-reference bevacizumab, MYL-1402O and US-reference bevacizumab, and between EU- and US-reference bevacizumab where least squares mean ratios of AUC_0–∞_ were close to 1, and 90% CIs were within the equivalence range (0.80–1.25). Secondary pharmacokinetic parameters (AUC from 0 to time of last quantifiable concentration [AUC_0–*t*_], peak serum concentration [*C*_max_], time to *C*_max_, elimination rate constant, and elimination half-life) were also comparable, with 90% CIs for ratios of AUC_0–*t*_ and *C*_max_ within 80–125%. Treatment-emergent adverse events were similar across all three treatment groups and were consistent with clinical data for bevacizumab.

**Conclusion:**

MYL-1402O was well tolerated and demonstrated pharmacokinetic and safety profiles similar to EU-reference bevacizumab and US-reference bevacizumab in healthy male volunteers. No new significant safety issues emerged (ClinicalTrials.gov, NCT02469987; ClinicalTrialsRegister.eu EudraCT, 2014-005621-12; June 12, 2015).

**Supplementary Information:**

The online version contains supplementary material available at 10.1007/s00432-021-03628-0.

## Introduction

The growth of blood vessels, a process known as angiogenesis, is essential for organ growth and repair (Carmeliet 2009; Folkman 2002). In cancer, angiogenesis is the mechanism required for tumor growth and metastasis (Folkman 2002). Vascular endothelial growth factor (VEGF) is a small signaling molecule that stimulates angiogenesis (Carmeliet 2009). Vascular endothelial growth factor-mediated angiogenesis is involved in invasive tumor growth and metastasis in cancers including nonsquamous non-small cell lung cancer (Han et al. 2001), colorectal cancer (André et al. 2000), breast cancer (Kurebayashi et al. 1999), cervical cancer (Hashimoto et al. 2001), and ovarian cancer (Nishida et al. 2004). Therefore, preventing VEGF-mediated angiogenesis is a therapeutic strategy to control cancer progression (Folkman 2002).

The biologic bevacizumab (Avastin^®^; Genentech, Inc, South San Francisco, CA) is a recombinant humanized monoclonal antibody that acts as a VEGF-specific angiogenesis inhibitor (Avastin 2019). Bevacizumab binds VEGF and prevents VEGF from interacting with Flt-1 and KDR receptors on endothelial cells, thereby inhibiting its biological effects. In combination with other anticancer therapies, bevacizumab has shown efficacy in the treatment of multiple cancer types (Roviello et al. 2017; Chen et al. 2014; Sandler et al. 2006; Garcia et al. 2020). In the United States and Europe, bevacizumab is approved in combination for the treatment of metastatic colorectal cancer; metastatic or recurrent nonsquamous non-small cell lung cancer; metastatic or recurrent cervical cancer; recurrent epithelial ovarian, fallopian tube, or primary peritoneal cancer; and metastatic renal cell cancer (Avastin 2019; Roche Pharma AG 2020). In addition, bevacizumab is approved as a single agent for the treatment of glioblastoma in the United States and in combination for the treatment of metastatic breast cancer in Europe. A meta-analysis performed on data from 38 clinical trials in bevacizumab-treated patients with solid tumors demonstrated a significant overall survival benefit compared with the control group (hazard ratio [HR] 0.92; 95% CI 0.88–0.95; *P* < 0.0001) in addition to a significant improvement in overall survival in colorectal cancer, cervical/uterine cancer, non-small cell lung cancer, and renal cancer (Roviello et al. 2017).

Manufacturing biologics is complex, which makes them expensive to produce (Rader 2008; Mellstedt et al. 2008). This in turn can limit patient access because of cost (Blackstone and Joseph 2013). As patents expire, biosimilars, which are structurally and functionally similar to the reference product (biologic), may increase patient access through lower costs (Mellstedt et al. 2008; Chopra and Lopes 2017).

Biosimilars have a different path to regulatory approval compared with small molecules. The US Food and Drug Administration (FDA) (FDA 2015) and the European Medicines Agency (EMA) (EMA 2015) have established guidance for the clinical development and regulatory approval of biosimilars. Biosimilarity with a reference product is defined for clinical and manufacturing purposes when “the biological product is highly similar to the reference product notwithstanding minor differences in clinically inactive components” and “there are no clinically meaningful differences between the biological product and reference product in terms of safety, purity and potency of the product” (EMA 2015; US Food and Drug Administration 2019). Currently, there are at least 16 biosimilars for bevacizumab under investigation, in addition to MYL-1402O (Liu et al. 2020; Busse and Lüftner 2019; Wang et al. 2019; Zhang et al. 2019), and two of these were recently approved by the FDA as bevacizumab biosimilars (Casak et al. 2018; Drug and Device News 2019).

The proposed biosimilar MYL-1402O has an amino acid sequence identical to bevacizumab. The similarity of MYL-1402O to bevacizumab was demonstrated in physicochemical analyses and nonclinical studies and MYL-1402O is currently being compared with bevacizumab in the first-line treatment of patients with stage IV nonsquamous non-small cell lung cancer. This publication presents the results of a phase 1 study in healthy adult male volunteers comparing the pharmacokinetic (PK) properties, safety, and tolerability of MYL-1402O with those of European (EU)-sourced reference bevacizumab and US-sourced reference bevacizumab, and those of EU- with US-reference bevacizumab.

## Methods

### Study design

**Fig. 1:**
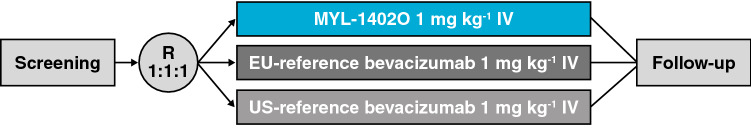
Study design: bioequivalence of MYL-1402O to EU and US-reference bevacizumab. IV, intravenous; R, randomization

This single-center, randomized, double-blind, three-arm, parallel-group study was conducted in healthy adult male volunteers from March 23, 2015, to November 5, 2015, at PRA Health Sciences in Groningen, The Netherlands (ClinicalTrials.gov, NCT02469987; ClinicalTrialsRegister.eu EudraCT, 2014-005621-12). After screening, eligible subjects were randomized (1:1:1) into three groups and received a single 1 mg kg^−1^ dose of MYL-1402O, EU-reference bevacizumab, or US-reference bevacizumab (Fig. 1). The dose was administered by intravenous (IV) infusion over 90 min, which is the recommended infusion duration for reference bevacizumab (Avastin 2019; Roche Pharma AG 2020). To seek approval in the European Union and United States, respectively, this three-arm trial was initiated after counseling with the EMA and FDA.

EU-reference bevacizumab and US-reference bevacizumab are usually administered at doses ranging from 5 to 15 mg kg^−1^ in patients with cancer (Avastin 2019; Roche Pharma AG 2020). Because the PK profile of bevacizumab is linear for doses ranging from 1 to 10 mg kg^−1^ (Roche Pharma AG 2020), the dose of 1 mg kg^−1^ was selected to minimize exposure in healthy volunteers. Healthy male volunteers were selected as the study population because of the influence of sex on the PK of bevacizumab (Avastin 2019; Knight et al. 2016). After correcting for body weight, clearance is approximately 26% higher in men than in women. More importantly, clinical study results have suggested that bevacizumab increases the risk of ovarian failure and may impair female fertility (Avastin 2019; Markus et al. 2017). The use of healthy volunteers minimizes the presence of factors that could confound the interpretation of PK results, such as varying tumor burden, comorbidities, complications from disease indications, and variations that could arise from a multidose regimen.

Healthy male subjects aged 18–55 years were eligible for inclusion in the study if they had a body mass index between 19.0 and 30.0 kg m^−2^ and weighed between 60 and 100 kg. All regular non-topical medication had to be stopped 30 days before admission to the clinic. Exclusion criteria included previous exposure to bevacizumab; history of severe allergic reactions to recombinant human or humanized antibodies; history of clinically relevant pathology or drug and/or food allergies; surgery (including dental procedures) within 28 days of study initiation and for ≥ 30 days after follow-up; medically significant dental disease or neglect; or history of bleeding disorders, thromboembolic conditions, gastrointestinal perforations or any fistulae, hypertension, orthostatic hypotension, fainting spells, or blackouts for any reason.

The primary objective was to compare the PK of MYL-1402O with that of EU- and US-reference bevacizumab and to compare the two reference products with each other after a single 1 mg kg^−1^ IV infusion over 90 min. Secondary objectives examined safety, tolerability, and immunogenicity of MYL-1402O and EU- and US-reference bevacizumab.

The study was approved by the independent ethics committee of the foundation Evaluation of Ethics in Biomedical Research (Assen, The Netherlands) and conducted in accordance with the general principles set forth in the International Ethics Guidelines for Biomedical Research Involving Human Subjects, International Council for Harmonization E6 Guideline for Good Clinical Practice, and the Declaration of Helsinki. Written informed consent was obtained from all subjects before study initiation.

### Study assessments

Study subjects entered the clinic on the afternoon of day -1 and left on day 9. Study assessments were performed on days 1 through 9 in the clinic and on days 12, 15, 22, 29, 43, 57, 71, and 85 during ambulatory visits. A variance of ± 1 day was allowed from day 22 onward, and a follow-up assessment was performed on day 99 ± 2 days. Serial blood sampling for PK analysis was collected predose and at the following times after the start of the infusion: hours 0.33, 1, 1.5, 2, 3, 4, 5, 6, 8, and 12 and days 2, 3, 4, 5, 6, 7, 8, 9, 12, 15, 22, 29, 43, 57, 71, 85, and 99.

Following the guidelines set forth by the FDA and EMA for biosimilar studies using intravenous administration and similar to the PK endpoints used in other bevacizumab biosimilar studies, the primary PK parameter assessed was the area under the serum concentration–time curve from 0 extrapolated to infinity (AUC_0–∞_) (EMA 2015; Hettema et al. 2017; Tajima et al. 2017; FDA 2016). Secondary PK parameters assessed were AUC from 0 to time of last quantifiable concentration (AUC_0–*t*_), peak serum concentration (*C*_max_), time of *C*_max_ (*t*_max_), elimination rate constant (*k*_el_), and elimination half-life (*t*_½_).

Safety and tolerability assessments consisted of adverse events (AEs), clinical laboratory parameters (clinical chemistry, hematology, coagulation, urinalysis), vital signs, 12-lead electrocardiogram (ECG), infusion site tolerance, physical examination, and immunogenicity. Adverse events were assessed throughout the study, and infusion site tolerance was assessed predose through 48 h after the start of the infusion or longer if needed until resolution of any event. A treatment-emergent AE (TEAE) was defined as any event not present before administration of study drug or any event already present that became worse in either severity or frequency after exposure to study drug. Severity of AEs was graded according to Common Terminology Criteria for Adverse Events (CTCAE) version 4.03, and events at the infusion site were scored according to a phlebitis scale. Vital signs (supine systolic and diastolic blood pressure, pulse, and respiratory rate) and 12-lead ECG were assessed during screening, day -1, at prespecified time points (vital signs: predose, days 1, 2, 3, 5, 9; 12-lead ECG: days 3 and 9), and at follow-up. Any clinically significant observations outside of the normal range for clinical laboratory tests, vital signs, 12-lead ECG, infusion site tolerance (only when grade ≥ 1), or physical examinations were recorded as AEs.

### Statistics

Sample size estimates were based on the following assumptions: the intersubject coefficient of variation (CV) of 20% for AUC_0–∞_, the 90% CI in the equivalence range of 0.80–1.25, the ratio of geometric means of any two treatment groups or any one pairwise comparison between treatment groups in the interval 0.95–1.05, and a 90% overall power for three pairwise comparisons (MYL-1402O vs EU-reference bevacizumab, MYL-1402O vs US-bevacizumab, and EU-reference bevacizumab vs US-reference bevacizumab). The above powering assumptions were determined by a small pilot study in which healthy adult male volunteers were administered 5 mg kg^−1^ of US-reference bevacizumab. The intersubject CV for AUC_0–∞_ for this pilot study group was found to be 16%. After 10,000 simulations, the upper bound of the 90% CI for intersubject variability for AUC_0–∞_ was found to be 20%, which was used as the basis for calculating the sample size. Similar intersubject variability has been reported for PK parameters in other studies comparing candidate bevacizumab biosimilars with reference bevacizumab (Knight et al. 2016; Zhi et al. 2011). Bioequivalence was concluded if the 90% CIs of the ratios (MYL-1402O/EU-reference bevacizumab, MYL-1402O/US-reference bevacizumab, and EU-bevacizumab/US-bevacizumab) of least squares means of the natural log-transformed AUC_0–∞_ (LNAUC_0–∞_) were bounded within 0.80–1.25. The PK for MYL-1402O, EU-reference bevacizumab, and US-reference bevacizumab were derived from the serum concentration–time curves, and the PK parameters were analyzed using analysis of variance (ANOVA). Statistical analysis was performed using the general linear models procedure (PROC GLM) of SAS^®^ Software (SAS Institute, Cary, NC). The model tested for treatment effects in the parameter means at an alpha level of 0.05. The PK parameters *t*_max_, *k*_el_, and *t*_1/2_ were analyzed using non-transformed data. Safety data were summarized using descriptive statistics.

## Results

### Subject demographics and baseline characteristics

Of the 181 subjects screened, a total of 111 (37 per treatment group) were enrolled and 110 (MYL-1402O, *n* = 37; EU-reference bevacizumab, *n* = 36; US-reference bevacizumab, *n* = 37) were included in the PK analysis (Online Resource 1). One subject who received EU-reference bevacizumab was excluded from the PK analysis set, before unblinding, because of anomalous elevations in serum bevacizumab concentrations observed at 10, 12, and 14 weeks postdose; exclusion of this subject did not affect the outcome of the PK analyses. Baseline subject characteristics were similar among treatment groups (Table 1). Most subjects were white (84%), and the mean (standard deviation [SD]) age of the total study group was 31 (12) years, with a mean body mass index (SD) of 24.4 (2.5) kg m^−2^. All 111 subjects completed the study and were included in the safety analysis.

**Table 1:** Subject demographic and baseline characteristics

Parameter	MYL-1402O (*n* = 37)	EU-reference bevacizumab (*n* = 37)	US-reference bevacizumab (*n* = 37)	Total (*N* = 111)
Age, mean (SD), year^a^	30 (11)	31 (13)	33 (12)	31 (12)
Male, *n* (%)	37 (100)	37 (100)	37 (100)	111 (100)
Race, *n* (%)				
White	34 (92)	30 (81)	29 (78)	93 (84)
Black	2 (5)	4 (11)	4 (11)	10 (9)
Asian	0	2 (5)	2 (5)	4 (4)
Multiple	1 (3)	1 (3)	2 (5)	4 (4)
BMI, mean (SD), kg m^−2^	24.0 (2.3)	24.5 (2.9)	24.7 (2.3)	24.4 (2.5)
Height, mean (SD), cm^a^	182 (6)	181 (8)	181 (7)	181 (7)
Weight, mean (SD), kg^a^	79.7 (9.0)	79.7 (9.3)	80.7 (9.2)	80.0 (9.1)

*BMI* body mass index, *SD* standard deviation

^a^Age, height, and body weight were determined at screening

Concomitant medications were used by 17 (15.3%) subjects during the study. These included medications taken for pain, inflammation, and influenza-like illness and were primarily analgesics, the majority being paracetamol. None of the medications were considered to influence the outcome of the study.

### Pharmacokinetics

**Fig. 2:**
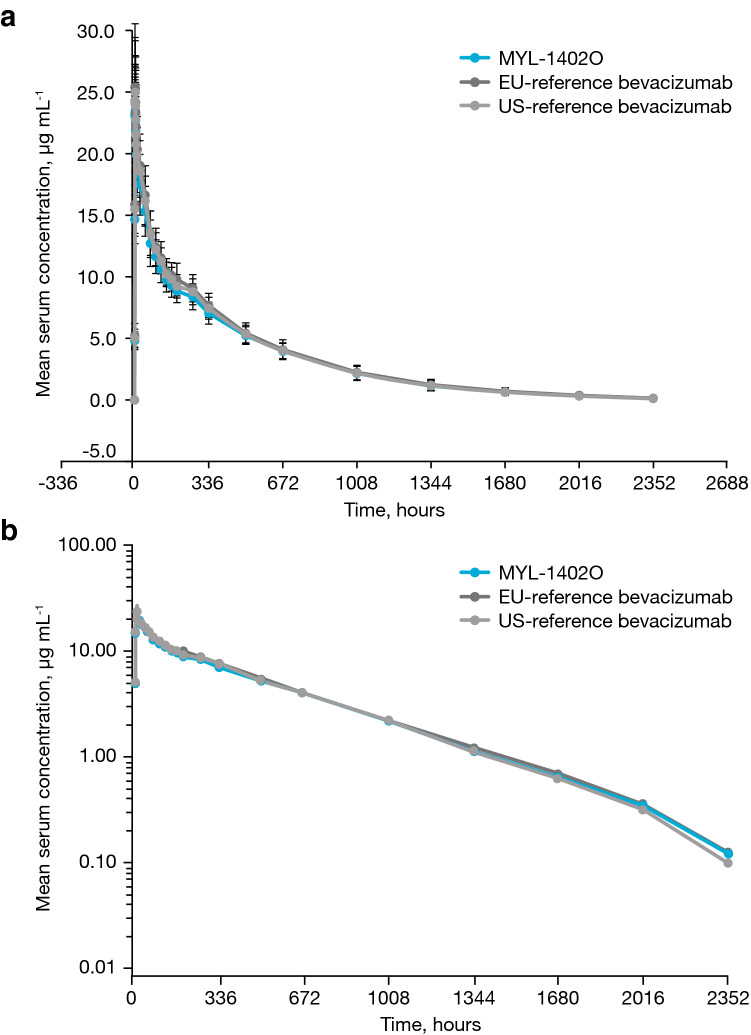
**a** Mean serum bevacizumab concentration vs time (linear scale). **b** Mean serum bevacizumab concentration vs time (semi-log scale). All treatments were a single intravenous dose of 1 mg kg^−1^ in 25 mL over 90 min

The mean serum concentrations of MYL-1402O and EU- and US-reference bevacizumab were similar throughout the study (Fig. 2). MYL-1402O and EU- and US-reference bevacizumab formulations demonstrated similar mean PK parameters and variability (Table 2).

**Table 2:** Summary of bevacizumab pharmacokinetic parameters

Parameter, mean (CV%)	MYL-1402O (*n* = 37)	EU-reference bevacizumab (*n* = 36)	US-reference bevacizumab (*n* = 37)
AUC_0–∞_, µg h mL^−1^	7663.6 (11.7)	8186.4 (15.1)	7904.2 (13.7)
AUC_0–*t*_, µg h mL^−1^	7526.5 (11.8)	8031.3 (14.8)	7764.8 (13.6)
*C*_max_, µg h mL^−1^	24.41 (11.5)	27.50 (18.7)	25.97 (13.0)
*t*_max_, h	2.533 (31.1)	2.338 (26.9)	2.798 (31.6)
*k*_el_, h^−1^	0.0019 (11.0)	0.0019 (15.2)	0.0020 (13.3)
*t*_½_, h	374.1 (11.3)	369.1 (15.0)	356.2 (14.0)

*AUC*_*0–∞*_ area under the serum concentration–time curve from 0 extrapolated to infinity, *AUC*_*0–t*_ AUC from 0 to time of last quantifiable concentration, *C*_*max*_ peak serum concentration, *CV* coefficient of variation, *k*_*el*_ elimination rate constant, *t*_*½*_ elimination half-life, calculated as 0.693/*k*_el_, *t*_*max*_ time of *C*_max_

### Primary and secondary PK endpoints

Bioequivalence was demonstrated between MYL-1402O and EU-reference bevacizumab, MYL-1402O and US-reference bevacizumab, and EU- and US-reference bevacizumab in pairwise comparisons (Table 3). The least squares mean ratios of the primary endpoint AUC_0–∞_ were close to 1, and 90% CIs were within 0.80–1.25 for all natural log-transformed AUC_0–∞_ comparisons. The AUC_0–*t*_ and *C*_max_ PK endpoints were also comparable across treatment groups with the 90% CIs for all ratios of AUC_0–*t*_ and *C*_max_ within 0.80–1.25. Secondary PK endpoints for *t*_max_, *k*_el_, and *t*_½_ were also similar across treatment groups.

**Table 3:** Summary of least squares means ratios and 90% CIs

Parameter	MYL-1402O/EU-reference bevacizumab	MYL-1402O/US-reference bevacizumab	EU-reference bevacizumab/US-reference bevacizumab
LNAUC_0–∞_, µg h mL^−1^	0.94 (0.8923–0.9898)	0.97 (0.9232–1.0233)	1.03 (0.9820–1.0893)
LNAUC_0–*t*_, µg h mL^−1^	0.94 (0.8931–0.9901)	0.97 (0.9230–1.0225)	1.03 (0.9812–1.0877)
LNC_max_, µg mL^−1^	0.90 (0.8490–0.9452)	0.94 (0.8921–0.9924)	1.05 (0.9955–1.1083)

*LNAUC*_*0–∞*_ natural log-transformed area under the serum concentration–time curve from 0 extrapolated to infinity, *LNAUC*_*0–t*_ natural log-transformed AUC from 0 to time of last quantifiable concentration; *LNC*_*max*_ natural log-transformed peak serum concentration

### Safety and tolerability

#### Adverse events

A total of 313 TEAEs were reported, 116 by 33 subjects (89%) who received MYL-1402O, 99 by 29 subjects (78%) who received EU-reference bevacizumab, and 98 by 28 subjects (76%) who received US-reference bevacizumab (Table 4). The most frequently reported TEAEs across all treatment groups were headache (20%), nasopharyngitis (12%), diarrhea (8%), and back pain (8%). Numerically higher reports of catheter site erythema and hematoma (both in the blood sampling arm) observed in the MYL-1402O group, compared with the EU- and US-reference bevacizumab groups, were not considered clinically relevant. There were no deaths, serious TEAEs, or discontinuations due to TEAEs. All TEAEs were either grade 1 (291/313 events in 87 subjects) or grade 2 (22/313 in 16 subjects) across all treatment groups. No systemic hypersensitivity or infusion reactions were observed.

**Table 4:** Most frequent treatment-emergent adverse events (≥ 5% of subjects)

*n* (%)	MYL-1402O (*n* = 37)	EU-reference bevacizumab (*n* = 37)	US-reference bevacizumab (*n* = 37)	Total (*N* = 111)
Subjects with ≥ 1 TEAE	33 (89)	29 (78)	28 (76)	90 (81)
Headache	7 (19)	9 (24)	6 (16)	22 (20)
Nasopharyngitis	6 (16)	2 (5)	5 (14)	13 (12)
Back pain	2 (5)	2 (5)	5 (14)	9 (8)
Diarrhea	3 (8)	2 (5)	4 (11)	9 (8)
Catheter site erythema (blood sampling arm)	5 (14)	3 (8)	0 (0)	8 (7)
Hematoma (infusion arm)	3 (8)	4 (11)	1 (3)	8 (7)
Abdominal pain	2 (5)	5 (14)	1 (3)	8 (7)
Myalgia	1 (3)	3 (8)	3 (8)	7 (6)
Catheter site pain (blood sampling arm)	3 (8)	3 (8)	1 (3)	7 (6)
Hematoma (blood sampling arm)	5 (14)	2 (5)	0 (0)	7 (6)
Pain in extremity	4 (11)	2 (5)	0 (0)	6 (5)
Dizziness	2 (5)	2 (5)	1 (3)	5 (5)
Paresthesia	0 (0)	3 (8)	2 (5)	5 (5)
Epistaxis	4 (11)	0 (0)	1 (3)	5 (5)

*TEAE* treatment-emergent adverse event

Forty-eight subjects (43%) reported 97 TEAEs that were considered related to one of the treatments by the study investigator. Across treatments, the most common treatment-related TEAEs (reported by ≥ 5% of subjects) were headache (16%; MYL-1402O, 16%; EU-reference bevacizumab, 22%; US-reference bevacizumab, 11%;), diarrhea (5%; MYL-1402O, 3%; EU-reference bevacizumab, 3%; US-reference bevacizumab, 11%), abdominal pain (5%; MYL-1402O, 3%; EU-reference bevacizumab, 8%; US-reference bevacizumab, 3%), and frequent bowel movements (5%; MYL-1402O, 3%; EU-reference bevacizumab, 5%; US-reference bevacizumab, 5%). Infusion site erythema considered by the investigator to be related to study treatment was reported in two subjects, both in the MYL-1402O group. One event each of catheter site pain and catheter site swelling related to EU-reference bevacizumab occurred. There were no clinically relevant findings with respect to clinical laboratory tests, vital signs, or physical examinations during the study. Hypertension and proteinuria were not observed in this study.

#### Immunogenicity

The percentage of subjects who tested positive for antidrug antibodies (ADA) throughout the study was comparable across the three treatment groups. Treatment-induced ADA positivity during the study was transient (Table 5). The potential impact of ADA on the PK parameters AUC_0–∞_, AUC_0–*t*_, and *C*_max_ was assessed by splitting subjects (*N* = 110) into low (*n* = 55) or high (*n* = 55) ADA groups based on ADA titer. When comparing the low and high ADA groups, the 90% CIs fell within the 80–125% bioequivalence limits for the three PK parameters (106.3–115.1%, 106.0–114.7%, and 101.2–110.7% for AUC_0–∞_, AUC_0–*t*_, and *C*_max_, respectively). Thus, any effect of ADA on AUC_0–∞_, AUC_0–*t*_, and *C*_max_ was not likely to be clinically relevant.

**Table 5:** Incidence of ADA by visit and treatment

Visit, *n* (%)	MYL-1402O (*n* = 37)	EU-reference bevacizumab (*n* = 37)	US-reference bevacizumab (*n* = 37)
Day 15	35 (95)	37 (100)	33 (89)
Day 43	28 (76)	28 (76)	31 (84)
Day 99	2 (5)	6 (16)	4 (11)

*ADA* antidrug antibodies

## Discussion

The proposed bevacizumab biosimilar MYL-1402O was bioequivalent to both EU-reference bevacizumab and US-reference bevacizumab when administered as a single-dose 1 mg kg^−1^ IV infusion over 90 min in healthy male subjects in this phase 1 study. EU-reference bevacizumab was also bioequivalent to US-reference bevacizumab.

This study used a parallel design because of the long half-life of bevacizumab, which was reported to be approximately 20 days (Avastin 2019; Roche Pharma AG 2020). Additionally, a parallel design allowed for comparison of the immunogenic potential of MYL-1402O with that of EU- and US-reference bevacizumab. This type of analysis would be prevented by multiple exposures in a crossover design study.

Bevacizumab PK are linear and predicted to reach more than 90% of steady-state concentration by 84 days. Population simulations of reference bevacizumab exposure show a median trough concentration of 80.3 µg mL^−1^ on day 84 after a dose of 5 mg kg^−1^ once every 2 weeks, and bevacizumab has a mean (CV%) clearance rate of 0.23 (33) L/day (Avastin 2019). ANOVA statistical analysis for the primary PK parameter, AUC_0–∞_, across all three treatment groups demonstrated that the 90% CIs of the ratios of geometric means ranged between 89.23% and 108.93% and were all within the predefined bioequivalence criteria of 80–125% for the natural log-transformed data. Furthermore, the secondary PK parameters, AUC_0–*t*_, *C*_max_, *t*_max_, *k*_el_, and *t*_½_, were also similar for each treatment group, with 90% CIs of the ratios for AUC_0–*t*_ and *C*_max_ falling within the predefined bioequivalence criteria.

The safety profiles of MYL-1402O, EU-reference bevacizumab, and US-reference bevacizumab were similar. The small differences in incidence rates between treatment groups are not considered to be clinically relevant and were probably due to small sample size for safety evaluation. All treatments were well tolerated, no infusion site events higher than grade 2 occurred, no serious TEAEs or discontinuations due to TEAEs were reported, and no clinically relevant findings for clinical laboratory parameters, vital signs, or physical examinations were observed.

In this population of healthy male adults, all TEAEs were mild or moderate in severity and consistent with the overall safety profile of bevacizumab based on clinical results from over 5700 patients treated with bevacizumab in combination with chemotherapy (Roche Pharma AG 2020). The most frequently observed adverse reactions across clinical trials in patients receiving bevacizumab were hypertension, fatigue, diarrhea, and abdominal pain (Roche Pharma AG 2020). In this study, volunteers were excluded if they had a history of hypertension. The most frequently reported TEAEs across all treatment groups in this study were headache (20%), nasopharyngitis (12%), diarrhea (8%), and back pain (8%), which are consistent with commonly reported TEAEs across all clinical studies of bevacizumab: diarrhea (> 10%), headache (1–10%), nasopharyngitis (1–10%), and back pain (1–10%) (Roche Pharma AG 2020). The safety and tolerability of the proposed biosimilar MYL-1402O, EU-reference bevacizumab, and US-reference bevacizumab were comparable to results from previous clinical studies of bevacizumab. The observed incidence of ADA, which was consistent across the treatment groups, was higher than the historically reported incidence, most likely because of the highly sensitive, drug-tolerant immunogenicity assay used in this study.

Results for MYL-1402O are similar to those observed for other bevacizumab biosimilars currently in clinical development, although it is important to note that these biosimilars were assessed using different administration schedules and doses in the linear range of bevacizumab PK. In healthy male volunteers, a single dose of 5 mg kg^−1^ of PF-06439535 demonstrated bioequivalence to both US-reference and EU-reference bevacizumab; TEAEs occurring in > 5% of subjects were upper respiratory tract infection, headache, dyspepsia, diarrhea, and tooth abscess (Knight et al. 2016). A single dose of 1 mg kg^−1^ of BI 695502 also demonstrated bioequivalence to US-reference and EU-reference bevacizumab in healthy male subjects, and the most common treatment-related TEAEs were upper respiratory tract infection and headache (Knight et al. 2016; Hettema et al. 2017). Bioequivalence to bevacizumab was demonstrated for BS-503a at a single dose of 3 mg kg^−1^ in healthy male volunteers, and the most common treatment-related TEAEs (observed in > 5% of subjects) were nasopharyngitis, headache, epistaxis, and rhinorrhea (Tajima et al. 2017). Finally, when administered at 15 mg kg^−1^ every 3 weeks for six cycles in patients with nonsquamous non-small cell lung cancer, ABP 215 and bevacizumab demonstrated equivalent efficacy and comparable safety profiles (Thatcher et al. 2016).

The current study used a dose of 1 mg kg^−1^, which is within the linear range of PK for bevacizumab, to limit exposure of a healthy population to a drug that is normally used to treat patients with cancer. Use of a subtherapeutic dose may limit the utility of the safety data; however, EMA guidelines for biosimilars state that subtherapeutic doses may be used in healthy volunteers (EMA 2015). Healthy volunteers were a suitable population for this study because they had few confounding variables that could cause major interindividual variation, allowing for the detection of subtle differences in PK profiles. MYL-1402O demonstrated bioequivalence in another PK study (Indian clinical trials registry identifier, CTRI/2014/11/005171) when compared with reference bevacizumab in 136 patients with metastatic colorectal cancer (mCRC) (Beniwal et al. 2017). Similar to the current study, no new or unexpected safety events were reported.

## Conclusions

In this phase 1 study, MYL-1402O, EU-reference bevacizumab, and US-reference bevacizumab were bioequivalent. ANOVA statistical analysis confirmed for all three pairwise comparisons that the 90% CIs of the geometric means for the primary PK parameter AUC_0–∞_ were within the predefined bioequivalence interval of 80–125%. The safety and tolerability of MYL-1402O, EU-reference bevacizumab, and US-reference bevacizumab were similar and consistent with prior clinical studies of bevacizumab.

## Supplementary Information

Below is the link to the electronic supplementary material.

Supplementary file1 (PDF 57 KB)

## Data Availability

Data available on request due to privacy/ethical restrictions. The data that support the findings of this study are available on request from the corresponding author. The data are not publicly available due to privacy or ethical restrictions.
