# Supplementary material for: A pharmacokinetics study of proposed bevacizumab biosimilar MYL-1402O vs EU-bevacizumab and US-bevacizumab
Source: J Cancer Res Clin Oncol. 2021 Apr 17;148(2):487–96. doi: 10.1007/s00432-021-03628-0 (PMC8800899; doi:10.1007/s00432-021-03628-0)

A Pharmacokinetics Study of Proposed Bevacizumab Biosimilar MYL-1402O vs EU-Bevacizumab and US-Bevacizumab

Journal of Cancer Research and Clinical Oncology

Matthew Hummel, Tjerk Bosje, Andrew Shaw, Mark Shiyao Liu, Abhijit Barve, Mudgal Kothekar, Mark A. Socinski, Cornelius F. Waller

Corresponding author:

Cornelius F. Waller, MD

Department of Hematology, Oncology and Stem Cell Transplantation

University Medical Centre Freiburg and Faculty of Medicine

University of Freiburg

E-mail: cornelius.waller@uniklinik-freiburg.de

Online Resource 1. Patient disposition. [a]Other reasons included outside of body mass index range, illness, failed physical examination, alcohol use within 48 hours of admission, medication use within 14 days of admission, consumption of >20 cigarettes in the 2 days before admission, use of cannabinoids 1 week before admission, inability to read/write properly, and other. Subjects who dropped out or withdrew for any reason, without successfully completing all screening evaluations, were considered screening failures.

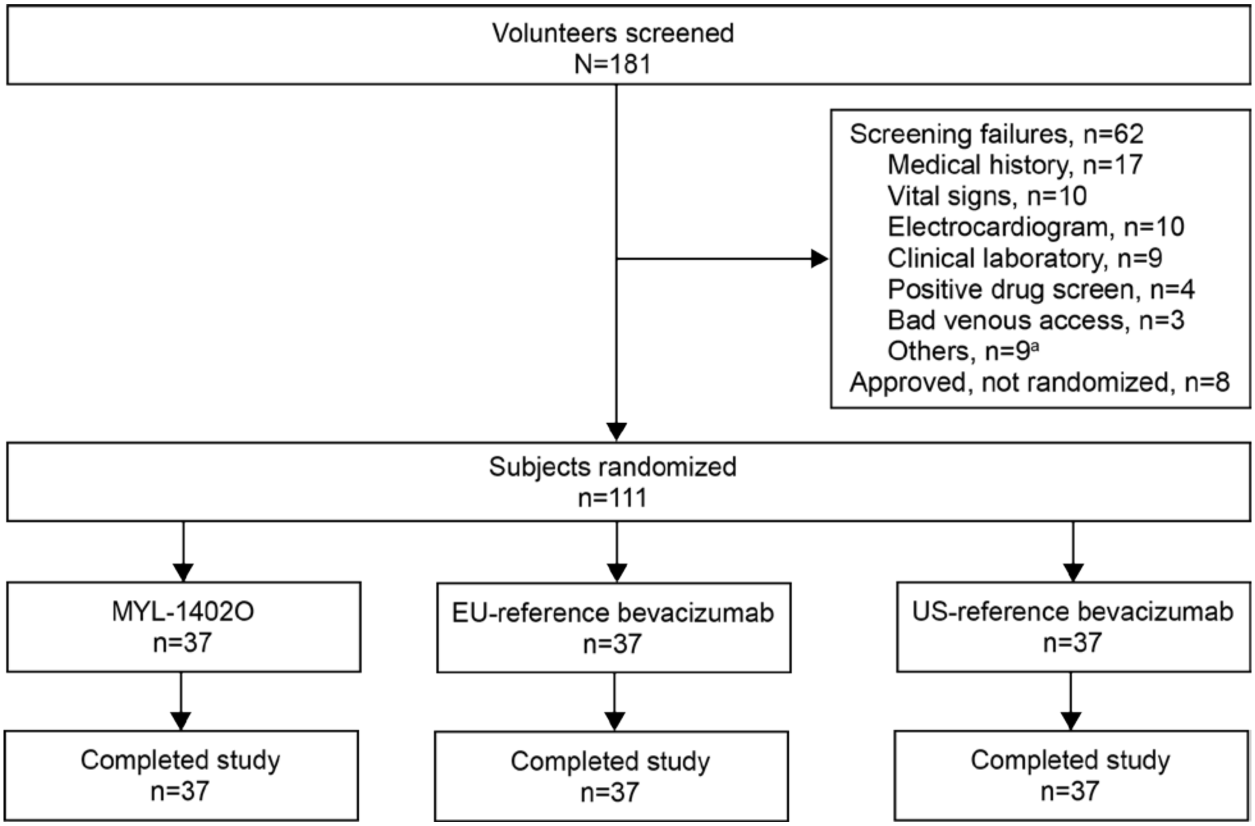

Supplement: Supplementary file 1 — Supplementary file1 (PDF 57 KB) [file 432_2021_3628_MOESM1_ESM.pdf]
